# Newer Ideas of the Causes and Treatment of Bright’s Disease

**Published:** 1907-02

**Authors:** Alfred C. Croftan

**Affiliations:** Chicago, Ill.


### SELECTIONS AND ABSTRACTS.

### NEWER IDEAS OF THE CAUSES AND THE TREATMENT OF BRIGHT'S DISEASE.

By Alfred C. Croftan, Chicago, Ill.

(Author's Abstract.)

The terms Bright's disease, nephritis and albuminuria are often employed synonymously. This is wrong. For in Bright's disease in the modern sense, the changes about the heart and the arteries predominate, and not infrequently precede the development of the renal signs. In all other forms of nephritis there are either no changes about the cardiovascular apparatus at all or they are demonstrably consecutive to the nephritis. Albuminuria finally is a sign that generally accompanies any form of nephritis but that may also occur without any real changes whatsoever.

High arterial tension with corresponding changes about the heart and arteries, then, is the determining feature of Bright's disease. The circulatory disturbances must be imagined to lead the nutritional disorders in a number of organs; and it is clear that those organs in particular are most apt to become affected that are supplied by end arteries. Chief among the latter are the kidneys, the retina and the brain; and as a matter of fact these three organs are most commonly involved in Bright's disease together with the cardiovascular apparatus.

In true Bright's disease the kidneys, as indicated, are by no means always involved first, although the first determinable signs may appear in the urine. If the dimensions of the heart and the blood-pressure were studied as carefully as a routine measure in every case as the urine is, (or should be) the cardiovascular changes would be found much more often before nephritic changes develop than is actually the case. This at least has been my experience!

It seems almost paradox to state that Bright's disease may occur without nephritis; but this is actually the case if we accept the newer conception of Bright's disease outlined above. It is altogether unfortunate that the term Bright's disease is retained in our medical nomenclature at all, for what Richard Bright originally described and what we understand by Bright's disease are radically different clinical entities.

The causes that determine the high blood-pressure and the cardiovascular changes that usher in every case of Bright's disease are still obscure. It is probable that manifold factors may become operative to produce this snydrome. I am an ardent adherent of the idea that circulating toxins must, in most cases, be incriminated with raising the blood-pressure; the toxins may be endogenous or exogenous in character, *i. e.* they may be introduced into the circulation from within by some deep seated perversion of the general metabolism or they may be introduced from without, *e. g.* from the gastrointestinal tract by the abnormal disassimilation of the gastric and intestinal contents.

The former event is probably less frequent than the latter; it is, moreover, less tangible and consequently not so easily remediable. In many cases a deep-seated hereditary element undoubtedly plays a commanding role, and here the metabolic side of the question urgently calls for elucidation. Some steps in this direction have been taken and a number of pressor principles have been isolated from among the intermediary produces of intracellular disassimilation. I call attention for instance to the purin bodies, precursors of uric acid, that possess marked pressure raising powers and that are capable, as I showed some years ago,[2] of producing many of the cardiovascular signs of Bright's disease in animals and also some of the renal changes.

In the bowel contents, on the other hand, a large number of pressor bodies have been found, especially when putrefactive processes are allowed to go unchecked. These bodies may directly enter the circulation in small quantities over long periods of time and produce chronic increase of the blood-pressure, or they may lead indirectly to intoxication of the liver cells that try to arrest them and thereby cause a condition of the hepatic insufficiency that in itself again leads to the flooding of the blood stream with a variety of abnormal products capable of causing high arterial tension and irritation of the kidneys.[3]

Treatment of Bright's disease must therefore concern itself with counteracting, so far as that is possible, the formation of these various groups of toxic bodies. This can be done by paying strict attention to the died and by promoting intestinal autisepsis by various means, the details of which cannot be discussed in this brief abstract. The best remedies for the latter purpose I have found to be the bile acid salts[4] and the sulpho-carbolate of zinc.[5] That intestinal putrefaction is being held in check can be determined by the reduction or disappearance of the aromatic sulphates (with the indican as their chief representative) from the urine, also from the disappearance of the conjucate glycuronates and the compound glycocolls. Bismuth subnitrate when given by mouth, together with intestinal antiseptics, moreover, should not color the stools black if intestinal putrefaction is being held in check.

The treatment of the small group of cases of Bright's disease that is not due to intestinal derangement but to metabolic perversions of an obscure type, usually occurring in young subjects, is much more difficult because nothing very tangible, in the obscurity of our present knowledge, presents itself for treatment.

In this group of cases one is limited in one's endeavors to purely symptomatic treatment. The latter, however, does not consist in the treatment of the renal complications alone, but more generally in the treatment of Bright's disease the renal idea should be largely relegated to the back-ground and attention is bestowed upon keeping down the blood-pressure and regulating the action of the heart. A case of Bright's disease should, broadly speaking, be treated as a heart case and not as a kidney case; for most sufferers from this disease die not from the nephritis but from failure of the heart and from the complications that are gendered thereby and that differ in no material respect from the secondary signs seen in any case of failing cardiac compensation.

The measures at our disposal for regulating the blood-pressure are dietetic, hydrotherapeutic and medicinal. The diet should be selected in such a way that no preformed pressor principles are ingested and the minimum of food is eaten that leads to the formation of such bodies; chief among these articles are nuclein containing foods and foods containing extractives, for the latter incorporate the purin bodies and the former lead to the formation of these purin bodies in process of intracellular disassimilation. Alcoholic beverages of all kinds should be eschewed. Tea and coffee and cocoa are also bad. Smoking is always detrimental.

Hydrotherapeutic measures are of signal value,[6] especially the simple warm bath every evening. The details of these measures cannot be described within the narrow frame of this abstract.

Of mdicines the best of all are the nitrites and preparations of nitroglycerin. They are rarely needed, however, if sensible hydrotherapy is instituted; and it is best to keep medicines in reserve for emergencies in this as in any other chronic disorder. The use of very small doses of digitalis, *i. e.* of one or two drops of a good tincture throughout the course of the dsorder, or at least for many months at a time, is of great value, inasmuch as the drug given in this way seems to regulate the heart's action, to protect it, one might imagine, from the abnormal stimulating effect of circulating toxins. The administration of such small doses even for long periods of time, in my experience, does not deprive the heart of a vigorous digitalis stimulation should it become necessary to use large doses in emergencies later on.

That in addition to all these measures the general nutrition of the patient should be kept up carefully by an almost mathematical regulation of the diet [1] and the ingestion of sufficient calories, need hardly be emphasized. Failure to do this, as for instance when patients with Bright's disease are placed for long periods of time on an exclusive milk diet[7] can, I believe, be incriminated with having destroyed many a sufferer from this disease. These victims of a dogma are literally starved to death and would probably have fared better had they adopted no dietetic regime at all. By this it is not intended to say that milk is not an excellent article of diet in this disorder, but it is not fitted for various reasons to be the exclusive pabulum for more than a few days at a time.

The psychic element in these patients finally must not be forgotten.

They should be taught to take a hopeful view of their condition in order that mental depression, worry or fear may not derange the digestive and assimilative functions of the liver[3] and in this way lead to a self intoxication that in any cardiovascular and cardiorenal disorder is to be strenuously avoided.

In regard to the so-called surgical treatment of Bright's disease, that has of recent years acquired some notoriety, I quote as follows from another article.[5]

Splitting of the kidney capsule, or decapsulation of the organ, for the cure of Bright's disease is altogether irrational. The temporary relief of tension may improve the blood supply to the kidneys, and hence restore for the time being some functional activity to diseased epithlia; and this improvement in the renal function may become manifest by a reduction of edema, by a transitory decrease in the albuminuria, the disappearance of formed elements (casts, etc.) from the urine, and an increase in the excretion of solids and of water.

Bright's disease, however, as we have seen in the preceding paragraphs, is a systematic disorder, and the nephritis is merely one of its symptoms. Any treatment of the kidneys alone, whether surgical or otherwise, is therefore purely symptomatic, and can in no sense be regarded as curative. One might as well amputate the rose spots in typhoid fever and expect to cure the disease.

It is not surprising to find, therefore, that no true case of Bright's disease has ever been permanently benefited by operations on the kidneys. The procedure is mentioned in this place merely to be condemned.

